# Time is of the essence: Exploring temporal and spatial organisation in episodic memory

**DOI:** 10.1177/1747021821993823

**Published:** 2021-02-25

**Authors:** Dan PA Clark, Davide Bruno

**Affiliations:** 1 Department of Psychology, Liverpool Hope University, Liverpool, UK; 2 School of Psychology, Liverpool John Moores University, Merseyside, UK

**Keywords:** Temporal, spatial, memory, episodic memory

## Abstract

There is disagreement in the literature as to whether episodic memory maintains an inherent temporal organisation, that is, whether learned items are necessarily organised along some temporal dimension or whether temporal organisation is a task-specific occurrence. The current series of experiments explored this issue. In Experiment 1, we tested whether temporal or spatial contiguity was present in an incidental encoding task where either strategy (but not both together) could be employed at test. In Experiment 2, we attempted to facilitate the use of a spatial retrieval strategy at test by asking participants to recall the location where target items had been displayed at study, after incidental encoding. Experiment 3 explored the role of study-test congruency by informing participants at encoding that they would be tested on either their memory for the temporal sequence or spatial locations, and then testing both at retrieval. Finally, Experiment 4 employed a masking task at encoding to ensure participants could not predict the true nature of the task, despite it being incidental, and a surprise free recall task. Predominantly, participants displayed recall performance consistent with temporal contiguity, although there was evidence for spatial contiguity under certain conditions. These results are consistent with the notion that episodic memory has a stable and predictable temporal organisation.

## Introduction

Questions about the way episodic memories are organised in the mind, if not the brain, have a long history (e.g., Mandler, 1967), with pre-eminent focus being paid to semantic structures and scripts (e.g., Schank & Abelson, 1977). However, when learning lists of semantically unrelated items, individuals are typically found to retrieve memories in the order they were experienced. This is commonly termed the temporal contiguity effect (e.g., Healey, 2018; Sederberg et al., 2010). The order in which semantically unrelated items are learned affects the probability of recall of these items (Murdock, 1962), with primacy (i.e., better recall for earlier items) and recency (i.e., better recall for later items) effects, but learning order also affects the sequence of retrieval (Sederberg et al., 2010). For instance, a number of studies have demonstrated that free recall of short lists (4–6 items) tends to begin with the first item and then commonly proceeds in a forward serial order, essentially following a temporal retrieval order (Neath & Crowder, 1996; Spurgeon et al., 2014; Ward et al., 2010). With longer lists, immediate free recall may often begin from the last presented item (Howard & Kahana, 1999), but as retrieval of one item can facilitate retrieval of items that were learned in nearby temporal positions (Kahana, 1996), temporal clustering in recall is frequently observed, regardless of starting position, list length or age of participants (Bruno et al., 2016; Talamonti et al., 2020). Therefore, temporal clustering/contiguity appears to be a central feature of episodic memory (Healey & Kahana, 2014; Kahana et al., 2008). Indeed, in a recent review, Healey et al. (2019) indicated that temporal contiguity is an important predictor of recall dynamics and while some factors affect the influence of contiguity, few eliminate it.

Despite the demonstrated importance of temporal retrieval in episodic memory, questions remain as to whether temporal contiguity is inherent in memory or rather a corollary of the way episodic memory is assessed. In a recent review, Hintzman (2016) has argued against the notion that temporal contiguity is an essential feature in episodic memory, suggesting that if participants can anticipate the method of testing, they will tailor their encoding strategy to suit the perceived testing paradigm, essentially maximising their own performance. According to Hintzman (2016), the majority of the literature supporting the ubiquitous presence of temporal retrieval in episodic memory has originated from studies employing multiple lists, which cue participants as to the nature of the experiment, and subsequently an intentional learning paradigm. In support of Hintzman’s (2016) claims, evidence points to examples of memory retrieval that are not temporal in nature. For instance, Curiel and Radvansky (1998, 2002) demonstrated increased use of temporal context information in map learning when place names were used, and increased spatial priming when participants were asked to point in the direction of locations. These findings support Hintzman’s suggestion that the nature of the testing conditions will influence the strategy used to retrieve information from memory. Similarly, Nairne et al. (2017) found no evidence of temporal clustering during a free recall task, except when participants were directly instructed to list the items in the order of presentation (see Experiment 3, but see Healey, 2018 for exceptions). These examples indicate that while temporal information can be employed as a successful retrieval strategy or at least, a successful retrieval cue in semantically unrelated lists, it is not the only retrieval method that can be utilised. Indeed, Polyn et al. (2009) suggest that at the time of learning, unrelated items are likely to form representational clusters based on either temporal or spatial types of information, which can then be employed as retrieval cues. In addition, Cortis Mack et al. (2018) demonstrated the coexistence of both spatial and temporal information in memory formation. Thus, if information can be organised as different representations (e.g., temporal and spatial) at encoding, then it is possible that both types of information will be stored in memory. Subsequently, any of these representations could be employed as a retrieval strategy at recall.

All in all, the literature remains mixed on the question of whether encoding the temporal contiguity together with learned information is a necessity, and we wish to tackle this question with the present article. Our driving hypothesis is that multiple representations, temporal and spatial in this case (Cortis Mack et al., 2018; Polyn et al., 2009), are possible for the same item, and that these representations both coexist and are inherent (see also McNamara et al., 1992). During retrieval, participants will then, either automatically or deliberately, activate one or both of these representations that will then cue retrieval of the rest of the sequence. The current series of experiments aimed to explore whether temporal contiguity is an inherent feature of memory retrieval using both incidental and intentional learning tasks over a single trial (c.f., Hintzman, 2016). Following Curiel and Radvansky (1998), our task can be completed successfully using either a temporal or a spatial strategy (Talamonti et al., 2020). In Experiment 1, we presented stimuli around a spatial array and observed whether temporal or spatial clustering was employed at retrieval in a surprise free recall task. In Experiment 2, we attempted to manipulate clustering again using a surprise test, but this time were presented it as spatial in nature. In Experiment 3, we looked at the effect of congruence between encoding instructions and expected testing task in spatial and temporal tasks. Finally, in Experiment 4, we adopted a simple spatial reaction time cover task at encoding and explored retrieval sequence using a surprise free recall task. As, typically, spatial and temporal information is conflated during study (e.g., left is processed before right), our experiments attempted to put participants in conditions where these types of information were pitched against one another, therefore essentially forcing participants to adopt either a temporal or spatial approach at recall.

## Experiment 1

### Methods

#### Participants

Thirty-one (7M: 24F) participants with a mean age of 20.35 years (*SD* = 5.92) took part in this study. Based on previous work conducted in our lab (Talamonti et al., 2020), where a similar study design was employed, the required sample size was calculated using an a priori power analysis. Using G*Power 3.1.7 (Faul et al., 2007) with an alpha = .05, power = 0.80, and an effect size *d* = 0.50, we obtained a required sample size of 27. Twenty-nine of these participants were right handed and two participants were left handed. All participants were either staff or students at Liverpool Hope University. All studies received ethical approval from the Liverpool Hope University ethics committee.

#### Design

The study had two, one-sample *t* test designs. The dependent variables were the temporal and spatial contiguity factors calculated from the retrieval sequence.

#### Stimuli and procedure

**Figure 1.**
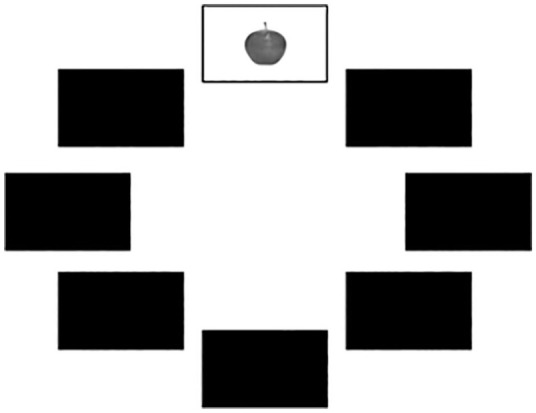
An illustration of the display stimuli. Here, the apple target is displayed in Location 1.

Participants were each shown eight targets, all of which were from the same semantic category and all were well known fruits (apple, banana, cherries, kiwi, lemon, orange, pear, and strawberry). Each of the targets was displayed in turn on a circular array comprising seven black boxes and a single box containing a target image (see Figure 1). Each of the boxes and target images was 200 × 200 pixels in size and was displayed on a screen resolution of 1,024 × 1,280 pixels. The order and location of the targets display were random, meaning any target could be displayed in any of the locations in any temporal order. The experiment was conducted using Eprime 2.0, and the experimental script recorded the order of display and the locations in which each target was displayed.

#### Encoding procedure

During encoding, all participants saw each target and each location only once. Participants were first displayed with a fixation cross for 500 ms, which was then replaced by the first target item to be displayed for 3 s before a 1-s interstimulus interval. This process was then repeated until all targets had been displayed. At encoding, participants were asked to complete a simply naming task and to say the fruit name aloud.

#### Retrieval procedure

In the retrieval phase, participants were provided with a response sheet and were asked to list as many of the target items that they had previously seen in any order. Participants were given a maximum of 3 min to complete this task (although it should be noted, most participants finished the task in under 1 min). Importantly, participants only completed the encoding task and retrieval task once.

### Measuring contiguity

#### Contiguity factors

As a result of the nature of the task, total recall performance was high (89%). This was by design as the central area of interest was the retrieval sequence, not overall recall. Interestingly, the first item displayed at encoding was the first item recalled in 67.74% of participants. As discussed in the procedure, each participant only completed a single encoding and a single retrieval task, however, participants could potentially retrieve the items following either a temporal or a spatial sequence. As such, the single output sequence was used to compute both temporal and spatial factors (a separate measure of temporal and spatial contiguity, respectively).

The first stage of calculating both spatial and temporal contiguity was to produce a recall matrix. Here, the participants’ retrieval list was compared with both the spatial and temporal encoding sequence. For instance, if the temporal sequence was A, B, and C, a recall sequence of 1, 2, and 3 showed the items recalled in the correct order. However, a sequence of 1, 3, and 2 showed the retrieval order of A, C, and B. The same approach was employed to generate the spatial sequences. This was generated for both the temporal and spatial sequences. Once the matrices were produced, the method of calculation for the temporal and spatial factor scores was replicated from Polyn et al. (2009) and Sederberg et al. (2010). As outlined in Sederberg et al. (2010), for each observed transition, the possible transition lags are ranked following the negative values of the serial position. To determine a factor score for that transition, the equation (*R* − 1) / (*N* − 1) was used, where *R* is the value of the rank for the observed transition and *N* is the number of possible transitions. The final contiguity score is simply the average of these values for all observed transitions. The resulting factor scores range from 0.0 to 1.0, where values greater than 0.5 suggest that participants were following the relevant (temporal or spatial) sequence and values below 0.5 suggest the sequence was not followed (Sederberg et al., 2010). This process was repeated for each participant’s spatial and temporal sequences. The factor scores were computed in bulk using the Behavioural Tool box release 1.01 for Matlab which was designed specifically for this purpose (Computational Memory Lab, 2020).

While the reporting of temporal factor scores is common in the literature, there have been some concerns raised about this approach. The primary issue here is that even in a randomly retrieved sequence, there would exist some contiguity between transitions, particularly in cases where there exists strong primacy or regency effects (Healey, 2018; Polyn et al., 2011). These artificial levels of contiguity can influence the results of the study. As such, we adopted the corrected measure of contiguity reported by Healey (2018). Here, as well as the temporal and factor scores for each participant, we also calculated the factor score for 10,000 random permutations of the sequence. Participant’s mean scores were then converted into *z* scores using the equation below

$$\frac{\left(Participants\;\bar{X}-Permutation\;\bar{X}\right)}{Permutation\;\;SD}$$

The *z* scores, *z* Temporal Contiguity and z Spatial Contiguity (zTC and zSC, respectively), now have an expected value of zero, so contiguity is present in when the condition average is significantly above zero.

#### Conditional response probabilities

As well as zTC and zSC scores, we also calculated lag conditional response probabilities (lag-CRPs). The lag-CRP is a measure of how a retrieved item follows another in the sequence (Howard & Kahana, 1999), providing a stronger representation of sequence than a serial position alone. Here, a positive lag indicates forwards recall and a negative lag is indicative of backwards recall, with lower lags suggesting performance closer to the sequence, in this case either the temporal or spatial sequences. In this study, lag-CRPs were calculated in accordance with Kahana (1996) and Sederberg et al. (2010) using the Behavorial Toolbox release 1.01 for Matlab (Computational Memory Lab, 2020). Lag-CRPs are calculated by dividing the number of times a transition of each lag size is observed by the number of times it could have been made. This excludes transitions to items which have previously been recalled or would fall outside the parameters of the list (for instance, assuming 8 items in a sequence and a starting point of Position 1, then there are seven possible transitions [transitions to Items 2, 3, 4, 5, 6, 7, and 8, with corresponding lags of +1, +2, +3, +4, +5, +6, and +7]; subsequently, in this case, no negative lags are possible) (Kahana, 1996).

### Results and discussion

**Figure 2.**
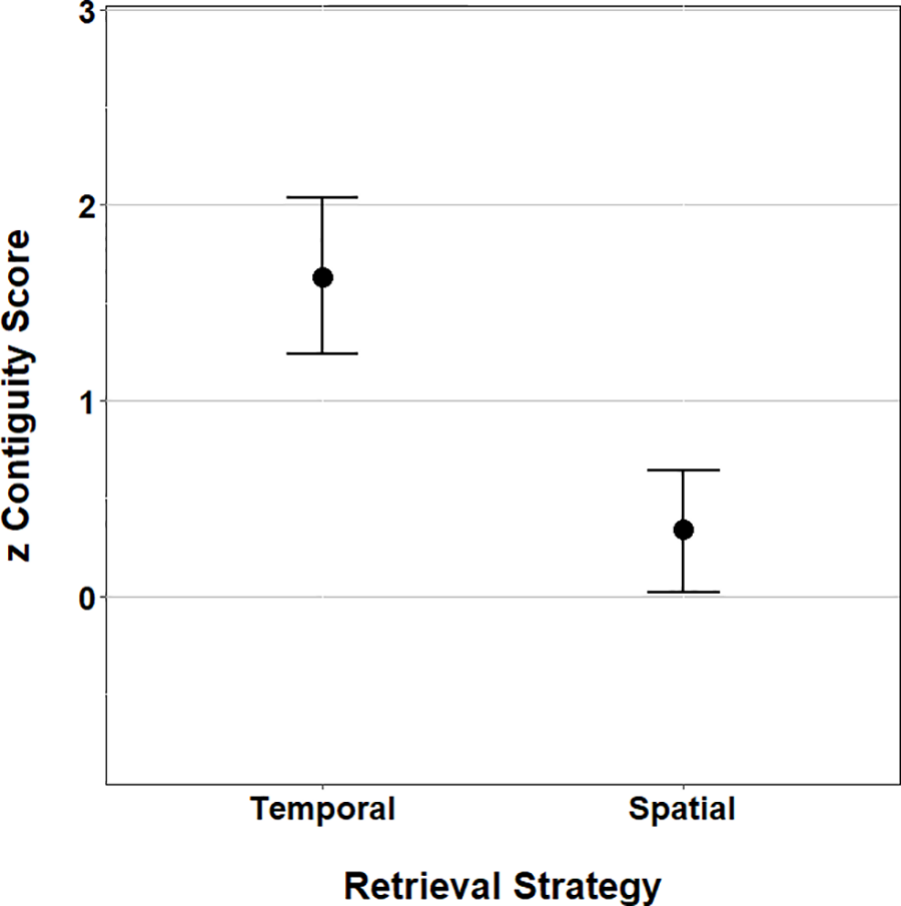
The mean scores zTC and zSC scores. Error bars represent ±95% CI.

The average zTC and zSC scores can be seen in Figure 2.

Figure 2 suggests the zSC score does not significantly differ from zero and subsequently does not differ to performance expected by chance (as indicated by the 95% confidence interval [CI]), whereas the zTC score suggests evidence of temporal contiguity. To formally test this observation, we conducted two, one-sample *t* tests to analyse whether the zTC and zSC scores significantly differed to chance (zero). The analysis demonstrated a significant effect of zTC scores, *t*(30) = 4.28, *p* < .001, *d* = 1.61, but no significant effect of zSC, *t*(30) = 1.60, *p* = .12.

**Figure 3.**
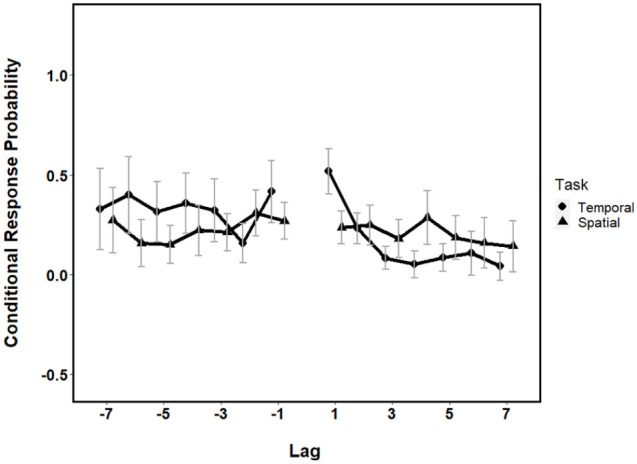
Conditional response probabilities as a function of lag for both the temporal and spatial retrieval strategies. Error bars represent ±95% CI.

To explore further which retrieval strategy is the best fit our data, we calculated lag-CRPs for both the temporal and spatial sequences. Figure 3 shows the lag-CRPs for both the temporal and spatial sequences. These data suggest that the temporal sequence produces higher CRPs for lower lag values, again providing evidence that a temporal retrieval strategy is the best fit of the data. In addition, Figure 3 suggests a higher proportion of positive lags, suggesting some use of a forwards retrieval strategy. This is consistent with previous literature (Kahana, 1996).

The aim of Experiment 1 was to explore whether participants engaged in a temporal or spatial retrieval strategy using a display which could facilitate both strategies, and with no explicit instruction to follow any specific encoding strategy. The results indicated that a temporal retrieval strategy demonstrated contiguity, suggesting that participants were more likely to follow the temporal strategy in a surprise free recall test. This finding was also supported further by the lag-CRP data. In addition, the analysis suggests that the zSC scores did not differ from chance. However, the test phase of Experiment 1 required participants to list all the study items with no specific constraint. It is possible that the retrieval instructions (i.e., “list the items you have seen”) may have facilitated the use of temporal clustering at this stage, despite the use of a spatial structure at encoding.

## Experiment 2

In Experiment 2, we attempted to encourage the use of an alternative, non-temporal, retrieval sequence at test by asking participants to retrieve not only the identity of the objects but also the locations in which each item was displayed. We predicted that when participants were required to draw on spatial information to complete the task, we would observe more reliance on a spatial sequence at retrieval.

### Methods

#### Participants

Twenty-two (6M: 16F) participants with a mean age of 21.73 years (*SD* = 5.40) took part in this study. Nineteen of these participants were right handed and three participants were left handed. All participants were naive to the true aims of the study and none had participated in Experiment 1. All participants were either staff or students at Liverpool Hope University.

#### Design

The study had two, one-sample *t* test designs. The dependent variables were the temporal and spatial contiguity factors calculated from the retrieval sequence.

#### Procedure

##### Encoding procedure

The encoding procedure in Experiment 2 was exactly the same as that in Experiment 1.

##### Retrieval procedure

To encourage a spatial retrieval procedure, participants were provided with a response sheet which replicated the display array (eight blank boxes displayed around a circle) from the encoding procedure. Participants were asked to fill in the array, writing the name of the target items in the location they were displayed. To keep a record of the order of recall, participants were also asked to indicate the recall position by marking the sequence with digits (i.e., 1 = first recalled, 2 = recalled second, and so on . . .).

### Results and discussion

**Figure 4.**
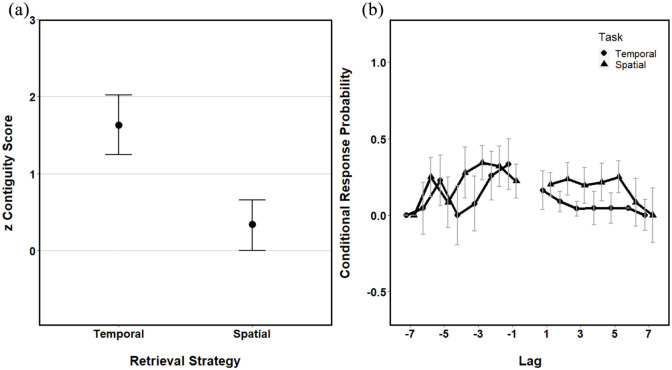
(a) The means factor scores for both a temporal and spatial strategy. (b) Conditional response probabilities as a function of lag for both the temporal and spatial retrieval sequences. All error bars represent ±95% CI.

As in Experiment 1, overall recall performance was high (88.6%, with 68.18% of recalling beginning with the first displayed item) so no further analysis was conducted on these data. To explore the central hypothesis, we calculated the zTC and zSC scores replicating the procedure outlined in Experiment 1. The mean factor scores for each strategy can be seen in Figure 4a.

Figure 4a shows that the zTC scores appear significantly larger than the zSC scores, which are only marginally above chance (0). Again, to test for contiguity, we conducted two, one-sample *t* tests. The results revealed that the zTC scores were significantly higher than 0 (chance), *t*(21) = 7.90, *p* < .001, *d* = 0.97, and the zSC scores did not differ significantly from chance, *t*(21) = 2.03, *p* = .06.

Again, lag-CRPs were calculated for both the spatial and the temporal sequences (see Figure 4b). These data suggest that the temporal sequence has a lower probability of selection for higher lag values (both positive and negative), whereas the spatial sequence probabilities appear consistent across all lag values. This is again indicative that a temporal sequence seems to be the best fit of these data. As with Experiment 1, the lag-CRPs appear to be higher for positive lag values, suggesting at least some use of a forwards retrieval strategy.

Experiment 2 aimed to encourage the use of a spatial retrieval strategy at test in an incidental learning task by asking the participants to retrieve the spatial location of targets. The results suggest that, despite location information being central to the retrieval task, data still support the predominance of a temporal retrieval strategy. This finding contrasts the views of Hintzman (2016) who argued that the nature of the retrieval task can influence the way information is recalled at test. It is, however, possible that because participants were only asked to consider the spatial information at retrieval, information had already been clustered temporally due to the nature of the encoding task. This question was addressed next.

## Experiment 3

In Experiment 3, we aimed to explore the effect of congruence between encoding strategy and expected testing method, on the use of spatial and temporal contiguity. To do this, we explicitly told participants at encoding that they were later to be tested for their ability either to retrieve items in a temporal order or to retrieve items in a spatial sequence.

### Methods

#### Participants

Forty (10M: 30F) participants with a mean age of 23.14 years (*SD* = 8.10) took part in this study. Thirty-seven of these participants were right handed and three participants were left handed. All participants were naive to the true aims of the study and none had participated in either Experiments 1 or 2. All participants were either staff or students at Liverpool Hope University.

#### Design

The study had a 2 × 2 mixed design with a single between-participants factor (encoding task, with two levels, temporal or spatial) and a single within-participants factor (sequence, with two levels, temporal and spatial). There were 19 participants in the temporal condition and 21 participants in the spatial condition. The dependent variables in this study were participants’ spatial and temporal contiguity scores.

#### Procedure

##### Encoding procedure

The encoding procedure in Experiment 3 was the same as that in Experiment 1. The participants saw each target fruit displayed in a random location for 3 s and were asked to name the item. The difference for Experiment 3 was that participants were told that they would be tested either on their ability to recall the stimuli in the order they were displayed or on their ability to recall the locations each object was displayed in.

##### Retrieval procedure

In the retrieval task, participants were asked to complete both the recall task from Experiment 1 (list the words in temporal order) and the recall task from Experiment 2 (complete the spatial array listing each item in the correct spatial location while recalling the order the locations were completed). The order of these tasks was counterbalanced across participants. In the temporal task, they were asked to recall the items in the order they were presented, whereas in the spatial task, they were asked to recall the items that were presented in each location starting with the target displayed at the top of the array and then to proceed clockwise around the array.

### Results and discussion

**Figure 5.**
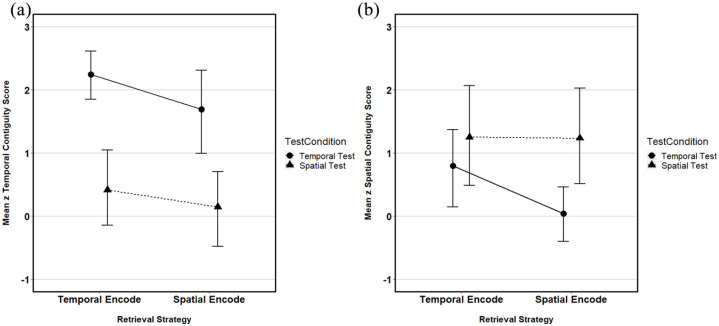
The mean *z* contiguity scores from both (a) temporal and (b) spatial sequences for all encoding and test combinations. All error bars represent ±95% CI.

**Figure 6.**
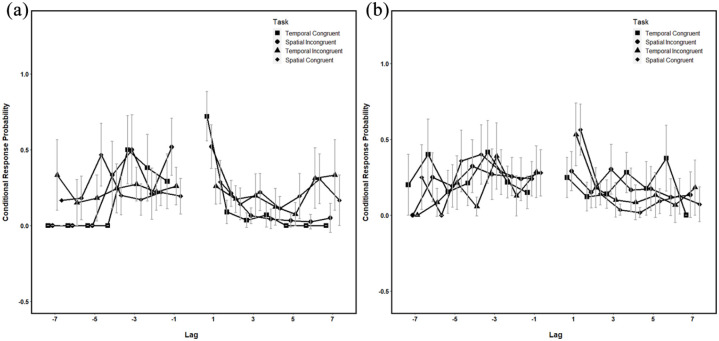
Conditional response probabilities as a function of lag for each encoding and retrieval combination for (a) the temporal and (b) the spatial retrieval sequences. Temporal congruent indicates a temporal encoding task and temporal retrieval task, temporal incongruent indicates temporal encoding task and spatial retrieval task, spatial incongruent indicates spatial encoding task and temporal test, and spatial congruent indicates a spatial encoding task and spatial retrieval task. All error bars represent ±95% CI.

Overall recall performance was high, 92.50% for the temporal encoding condition and 86.81% in the spatial encoding condition. Again, the first item retrieved was predominantly the first item displayed at encoding (81.25%). Once data were collected, the first stage of analysis was to calculate the zTC and zSC scores for both retrieval strategies and both encoding conditions, in addition to the lag-CRPs, for each of the retrieval strategies in both encoding conditions. As we deemed the spatial and temporal contiguity scores separate dependent variables, we explored each in separate analyses. The mean contiguity scores can be seen in Figure 5, and lag-CRPs for each strategy can be seen in Figure 6.

Hintzman (2016) argued that if participants are able to predict how they will be tested, they will adopt a memory strategy to maximise performance at retrieval. Following this argument, when participants expect a temporal recall task, then the temporal retrieval strategy should yield higher levels of contiguity than a spatial retrieval strategy, and vice versa. It is less clear, however, what to expect when the learning and test instructions are incongruent. In Experiment 2, we implicitly attempted to encourage and observe a spatial strategy; nevertheless, we still observed that temporal clustering was dominant. Therefore, we predict the same pattern in Experiment 3: temporal clustering will win over spatial clustering, except when spatial instructions are consistently provided both at study and test. As our central hypothesis is concerned with whether we observed higher levels of contiguity within the congruent strategy to encoding, we examined the differences between zTC and zSC separately.

#### Temporal contiguity analysis

The first stage of the analysis was to explore the standardised temporal contiguity scores for all the encoding and retrieval strategies. These can be seen in Figure 5a. These data met parametric assumptions, and as such, a 2 × 2 mixed analysis of variance (ANOVA) with a single between-participants independent variable, encoding scenario (with two levels, temporal or spatial), and a single within-participants independent variable, retrieval task (with two levels, temporal and spatial), was employed to explore these data. Results showed a significant main effect of retrieval task, *F*(1, 38) = 27.19, mean square error (MSE) = 2.08, *p* < .001, $\eta_{p}^{2}$ = .42, but no significant main effect of encoding task, *F*(1, 38) = 2.17, MSE = 1.57, *p* = .149, or interaction, *F*(1, 38) = 0.189, MSE = 2.08, *p* = .666.

These findings are supported by the CRP data, which indicate the highest probability of *a* + 1 transition in both the temporal retrieval conditions irrespective of encoding task (see Figure 6a), although *a* + 1 transition was also the most likely transition in the spatial retrieval condition. Together, these data suggest that when participants are tested for temporal order, they accurately recall the temporal sequence irrespective of whether they expected a temporal or spatial test at encoding. However, when they are tested on the spatial sequence, they show no evidence of temporal contiguity.

#### Spatial contiguity analysis

A similar pattern of results was observed when exploring the standardised spatial contiguity scores. Figure 5b shows the *z* spatial contiguity scores for both encoding conditions and both retrieval conditions. Again, these data met parametric assumptions and were analysed using a 2 × 2 mixed ANOVA. The analysis demonstrated a significant main effect of retrieval task, *F*(1, 38) = 4.75, MSE = 2.85, *p* = .036, $\eta_{p}^{2}$ = .11, but again no significant main effect of encoding task, *F*(1, 38) = 1.48, MSE = 1.99, *p* = .231, or an interaction, *F*(1, 38) = 0.937, MSE = 2.85, *p* = .339.

The lag-CRP results mirrored those reported in the temporal analysis (see Figure 6b): there was a higher likelihood of *a* + 1 spatial transition in both spatial retrieval tests, again irrespective of how expected test at encoding. These results suggest that participants are more likely to follow the spatial sequence when specifically asked to. In addition, when tested on the temporal sequence, participants did not demonstrate significant spatial contiguity, but it should be noted that with a temporal encoding task and temporal retrieval, participants did demonstrate spatial contiguity above that expected by chance (zero), although this analysis did not demonstrate a significant interaction.

Experiment 3 aimed to test whether the anticipation of test type influenced participants to adopt a particular memory strategy to maximise performance (measured by temporal and spatial contiguity). This study demonstrated a congruency effect when the test matched what was expected at learning: this is consistent with our prediction that a match between study and test instructions should favour the matched clustering modality. However, the current data also show that participants can demonstrate spatial contiguity when the incongruent temporal test is expected and temporal contiguity when anticipating the incongruent spatial encoding task. We therefore draw mixed conclusions from these results. First, Hintzman’s (2016) suggestion that when participants can anticipate the method in which they will be tested, they will adopt an appropriate strategy at encoding is partially supported, as participants demonstrated temporal contiguity and spatial contiguity in the respective congruent tests. However, we did also observed temporal contiguity following spatial encoding and spatial continuity following an anticipated temporal test which contrasts with Hintzman’s predictions. Overall, as reported elsewhere in the literature (Cortis Mack et al., 2018), our findings support the notion that spatial and temporal context information coexists in memory and can be activated even when incongruent to the encoding instructions. We addressed the issue of the incidental paradigm further in Experiment 4.

## Experiment 4

Experiments 1–3 all made use of the same encoding cover task, which was to name the item displayed. However, as previously discussed, Hintzman (2016) argues that if participants can determine how they will be tested, this will influence the retrieval process employed. It is conceivable therefore that in Experiments 1–3, some participants may have determined the true nature of the study, which in turn would have emphasised the use of temporal contiguity. Therefore, in Experiment 4, we used a cover task: we instructed participants to focus on clicking onto the stimuli as fast as possible and that we were interested in their reaction times. This way, the true scope of the research was hidden to participants.

### Methods

#### Participants

Forty participants (20M: 20F) took part in this study. All participants were undergraduate students and had a mean age of 20 years. Some participants volunteered in exchange for partial course credits.

#### Design

The study had two, one-sample *t* test designs. The dependent variables were the temporal and spatial contiguity factors calculated from the retrieval sequence.

#### Encoding procedure

During encoding, all participants were informed that they were taking part in a simple reaction time experiment. The stimuli were displayed in the same way as in Experiments 1–3; however, instead of naming the fruits, participants were asked to click the mouse to highlight the fruits’ location as quickly as possible. All fruits were displayed for 3 s, regardless of reaction time speed, and then, the mouse cursor was automatically re-set to the centre of the array before the next trial took place. Participants saw each item and each location in a random order.

#### Retrieval procedure

In the retrieval phase, participants were provided with a response sheet and were asked to list as many of the target items they had previously seen in any order. Participants were given a maximum of 3 min to complete this task (although it should be noted, as in the previous tasks, most participants finished the task in under 1 min). Importantly, participants only completed the encoding task and retrieval task once.

### Results

**Figure 7.**
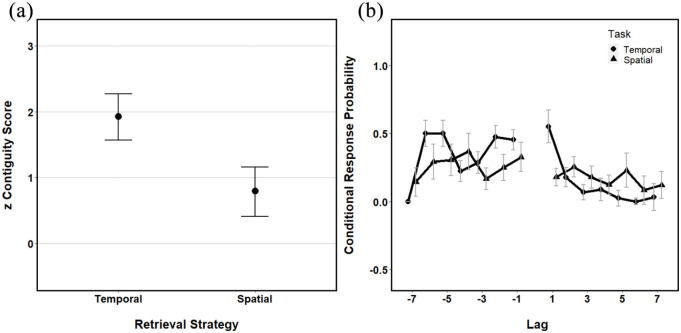
(a) The mean *z* contiguity scores from both temporal and spatial retrieval sequences. (b) Conditional response probabilities as a function of lag for both the temporal and spatial retrieval sequences. All error bars represent ±95% CI.

Overall recall performance was again high with 85.95% of items being recalled. Again, the recall sequences were used to calculate the zTC and zSC scores and in addition, the lag-CRPs for each strategy. These can be seen in Figure 7.

Figure 7 shows that, while both scores significantly differ from 0 (as indicated by the 95% CI), there appears to be significantly higher temporal contiguity than spatial contiguity. A one-sample *t* test revealed that there was a significant effect of zTC, *t*(39) = 11.04, *p* < .001, *d* = 1.08, and a significant effect of zSC, *t*(39) = 3.61, *p* = .001, *d* = 1.21. The mean contiguity scores suggest that there is higher contiguity for the temporal sequence. This finding is mirrored by the lag-CRPs which show the highest likelihood of a one-step forward transition in the temporal condition.

Experiment 4 aimed to explore the temporal contiguity effect using an alternative encoding cover task which would encourage participants to adopt a spatial retrieval strategy. While we found some evidence for spatial contiguity, which suggests that a spatial cover task might encourage spatial contiguity at retrieval, this was far smaller than the evidence of a temporal contiguity effect, further supporting the idea that temporal contiguity is an inherent feature of episodic memory.

## General discussion

The present article tested whether temporal contiguity, along with other forms of information (i.e., spatial locations), is an inherent feature of our memory systems. The current experiments demonstrate that participants produce patterns of recall that are generally more consistent with employing temporal contiguity at retrieval, as opposed to spatial contiguity, including when the retrieval task is designed to promote the use of the latter (Experiment 2). However, we did find evidence of spatial contiguity in some circumstances. Experiment 3 showed that participants had the ability to retrieve the items following a spatial sequence if instructed to do so. More interestingly, we found that when an encoding task was employed to attempt to promote spatial contiguity, we found some evidence of it in an incidental learning task (Experiment 4). However, while we demonstrated some spatial contiguity, this was far lower overall than the levels of temporal contiguity observed following the same spatial cover task. Collectively, these results suggest that temporal contiguity may be a characteristic principle of episodic memory (Hurlstone et al., 2014), which is consistently encoded together with the information and, at least when semantic clustering is not available, is prioritised as a prompting cue when retrieving information.

Consistent with previous findings with short word lists (e.g., Ward et al., 2010; but note that Cortis et al., 2015, found that word lists of eight items began roughly equally from Item 1 or from the last four), we observed the tendency to initiate recall from the beginning of a list, and then move forward. Subsequently, our results suggest that the first item may typically be most memorable in short lists, appealing to a primacy effect, rather than a recency effect. The former has traditionally been attributed to the fact that items presented early on a list are granted more opportunities for rehearsal, and thus can be memorised better. However, recent reports have suggested rehearsal may not be sufficient to explain primacy effects in short lists (Grenfell-Essam et al., 2013; Spurgeon et al., 2014). Excluding rehearsal, then a possible explanation of our results, also previously presented in Bruno et al. (2016, 2020), relies upon a combination of memorability and attributional cueing. Once the most memorable item is retrieved (i.e., often the first and most distinctive perhaps due to increased attentional focus on that item (Sederberg et al., 2006), so-called “edge effects” (Brown et al., 2007), or greater availability of processing resources (Tulving, 2008)), it is then possible that features of this item, such as temporal and/or spatial cues, will facilitate retrieval of other items and therefore influence the sequence of recall. This notion is consistent with Underwood (1969], who argued that “. . . strength, so called, is a bi-product of the attributes, of which the temporal attribute is only one” (p. 560), but also broadly consistent with temporal context models (e.g., Howard et al., 2005; Howard & Kahana, 1999; Howard & Kahana, 2002), which posit that retrieval of an item activates its encoding context, thus facilitating subsequent retrieval of items that share that (temporal) context (i.e., items learned around the same time). In this case, temporal or spatial contiguity would emerge depending on the prevailing cue, or attribute, and so it becomes important to be able to establish what may affect cue strength, and not just item strength.

The findings of the current studies support the notion that our memories present a stable and predictable temporal organisation (Healey & Kahana, 2014; Kahana et al., 2008). However, we have also demonstrated that the spatial sequence can be retrieved under certain conditions. Although, it is important to note the observed spatial contiguity only exceeded that observed for temporal contiguity when the participants were specifically instructed to follow that sequence. In incidental learning conditions, with retrieval not guided by the experimenter, we found stronger evidence for temporal contiguity. This conclusion raises questions as to why temporal contiguity seems to be favoured over spatial contiguity in our data. One possibility is that temporal information is generally better encoded than spatial information, resulting in stronger representations for the former. However, this explanation does not fully explain the pattern of results observed in the current experiments because in Experiment 3, participants demonstrated a benefit for a spatial strategy over a temporal strategy, even when they expected a temporal test. The match between encoding and testing conditions, or transfer-appropriate processing (Morris et al., 1977), should be expected to yield stronger representations for either type of information, whereas we found that participants could recall both sequences, regardless of the encoding instructions when specifically instructed to do so. Therefore, either the expectation that matching encoding and testing method should produce a stronger memory trace is incorrect, which would be inconsistent with a vast swath of the literature and with our finding that there was also a temporal benefit when a temporal task was expected, or perhaps temporal information is the stronger or more distinct of the two attributes or contextual cues, regardless of expectation. However, this primacy of temporal information over spatial information may only be true when processing verbal stimuli. While we used images as targets, the naming task in Experiments 1–3 could encourage participants to treat them as verbal stimuli. Indeed, Curiel and Radvansky (1998, 2002) have demonstrated increased use of temporal information when place names (linguistic information) were introduced in a memory task, but increased spatial priming when names were omitted and responses were indicated using a pointing task. This finding appears to suggest that our pattern of results may be unique to language-based tasks—perhaps due to the sequential nature of sentence construction. However, in Experiment 4, we did not employ the naming task, rather a spatial reaction time measure, and we still observed higher levels of temporal contiguity (although we did also observe spatial contiguity at lower levels). This would suggest that the naming task leading to verbal encoding cannot fully explain our findings.

As our focus in this article was to examine temporal and spatial contiguity in free recall, semantic relations between items were controlled by using the same semantic category (fruit). Indeed, we do expect that semantic clustering would provide a stronger encoding environment than temporal clustering. This assumption is supported by Nairne et al. (2017), who reported no evidence of unprompted temporal clustering in a free recall task when participants were required to rate the survival properties of the items at encoding. It is likely, in this case, as expected, that semantic clustering would have over-shadowed temporal clustering, thus rending the latter cue much weaker.

One limitation of the current set of studies is that to be able to isolate the temporal and spatial sequences, we could not display targets with spatial continuity (i.e., starting at the top location and sequentially following a clockwise spatial sequence around the array). If items had been presented in this fashion, it would have been impossible to ascertain whether spatial or temporal clustering were more likely, as both sequences would produce the same deviation, regardless of the strategy employed. It is possible that by not following a directly logical spatial sequence, the current design would promote the use of a temporal retrieval strategy. We do not think that this limitation invalidates the current findings, especially in Experiment 3 when participants were told to encode the spatial locations for subsequent test; however, it does raise questions about how to encourage the use of a spatial strategy without confounding temporal and spatial sequence. Future studies should attempt to address this issue. A further limitation here is that of list length. In this study, we explored the contiguity effect using eight target items. As previously noted, shorter list lengths may encourage participants to begin with the first displayed target item (e.g., Ward et al., 2010) and longer lists may encourage participants to start with retrieval of the last displayed item (Howard & Kahana, 1999). In addition, Healey et al. (2019) reported that temporal contiguity is more likely in shorter lists. This is a potential issue; however, again, we do not believe it invalidates the current findings. Simply because the free recall of the sequence starts with the first displayed target, it does not imply that subsequent items must be retrieved following a temporal sequence. Indeed, in Experiments 2 and 4, we employed encoding instructions that aimed at facilitating retrieval following a different sequence, yet continually found evidence of temporal contiguity. This contrasts the prediction of Hintzman (2016) who suggested that if participants did not anticipate a temporal retrieval task, they could use alternative strategies to guide retrieval. However, list length is an important factor and should be explored in subsequent work. In addition, there are two further limitations which should be considered. The first is that while Experiments 1, 2, and 4 all employed an incidental learning paradigm, we did not survey the participants after the experiment was complete to ensure that they had remained naive to the true aims of the study at encoding. Indeed, the finding of spatial contiguity in Experiment 4 could be potentially explained if the participants had anticipated a spatial memory task following the reaction time encoding. We do not think this invalidates the current findings but it does encourage further testing of the effect. In addition, the spatial testing task employed in Experiments 2 and 3 asks participants to retrieve the locations but manually record their temporal order as they did. This retrieval task could draw attention to a desire to track temporal order and subsequently yield an experimenter demand characteristic. We do not believe this to be the case, especially as the same test task was used in Experiment 3 when spatial contiguity was demonstrated even when incongruent to the expected test at encoding. However, the tracking of spatial and temporal sequence should be considered and any potential confound designed out in subsequent experiments.

A final issue that should be considered is the reported contiguity scores in the current experiments. The reported values in this study are substantially larger than those reported elsewhere in the literature (e.g., Healey, 2018). Because all calculated contiguity scores were done using the Computational Memory Lab’s (2020) Behavioural Tool box release 1.01 for Matlab, we do not believe that this is a result of a differing method of calculation. As such, these differences require further consideration. One explanation is the type of stimuli employed, we used a single 8-item list and all our target items were pictures from the same semantic category. Healey (2018), in contrast, used larger lists (16 items) of predominantly semantically unrelated lists (randomly drawn from a pool of 1,638 words). As there is substantial evidence that pictures are recalled better than words (Paivio, 1971; Rajaram, 1996) and that items with a shorter list length commonly follow a temporal order (Neath & Crowder, 1996; Spurgeon et al., 2014; Ward et al., 2010), these differences in magnitude of the effect may be accounted for by these methodological differences. In addition, while we believe we had the necessary power to observe the reported effects, we note that others have had substantially larger sample sizes (see Healey et al., 2019). We also note that in our experiments, where temporal contiguity was explored in conditions of incidental learning, we see high consistency in the values for contiguity (zTC values between 1.5 and 2.0, which would overlap in the respective CIs, though these were higher in the intention learning paradigm used in Experiment 3), despite changes in methodology. This again supports the notion that our findings represent a reliable effect and not a methodological flaw. However, it would be useful to systematically investigate how these methodological changes affect the observation of contiguity scores to help inform future work.

In sum, the current studies aimed to explore whether temporal contiguity is an inherent feature of memory which later influences subsequent retrieval processes. While we detected some evidence for spatial contiguity, all four experiments indicated recall performance that supports an inherent functional temporal organisation in memory.
